# Supplementary material for: Hybrid Models and Biological Model Reduction with PyDSTool
Source: PLoS Comput Biol. 2012 Aug 9;8(8):e1002628. doi: 10.1371/journal.pcbi.1002628 (PMC3415397; doi:10.1371/journal.pcbi.1002628)
Supplement: Text S4 — Complete source code for the PyDSTool package (version 0.88.120504). Includes API documentation and help files linking to web pages. This file is identical to the current public release on Sourceforge.net. (ZIP) [file pcbi.1002628.s004.zip › PyDSTool/html/PyDSTool.Toolbox.optimizers.helpers.quadratic.Quadratic-class.html]

xml version="1.0" encoding="ascii"?


PyDSTool.Toolbox.optimizers.helpers.quadratic.Quadratic


| Home | Trees | Indices | Help | PyDSTool |
| --- | --- | --- | --- | --- |

Package PyDSTool ::
Package Toolbox ::
Package optimizers ::
Package helpers ::
Module quadratic ::
Class Quadratic

[hide private]

[frames] | no frames]

# Class Quadratic

source code

```
object --+
         |
        Quadratic
```

Known Subclasses:
:   - levenberg\_marquardt.LMQuadratic

---

Defines a cost function with a quadratic cost


|  |  |
| --- | --- |
| Instance Methods | [hide private] |

|  |  |
| --- | --- |
| \_\_init\_\_(self, x, y, f)  Creates the function : - x is the parameters for the function - y is the data to approximate - f is an object function defining the gradient and the Hessian if needed It takes two parameters when called, the values x where the function should be computed and an array params that contains the parameters. | source code |

|  |  |
| --- | --- |
| \_\_call\_\_(self, params)  Computes the cost for the specified parameters | source code |

|  |  |
| --- | --- |
| gradient(self, params)  Computes the gradient of the function | source code |

|  |  |
| --- | --- |
| hessian(self, params)  Compute sthe hessian of the fit function | source code |

**Inherited from `object`**:
`__delattr__`,
`__getattribute__`,
`__hash__`,
`__new__`,
`__reduce__`,
`__reduce_ex__`,
`__repr__`,
`__setattr__`,
`__str__`


|  |  |
| --- | --- |
| Properties | [hide private] |

**Inherited from `object`**:
`__class__`


|  |  |
| --- | --- |
| Method Details | [hide private] |

|  |  |
| --- | --- |
| \_\_init\_\_(self, x, y, f)  *(Constructor)* | source code |

```
Creates the function :
- x is the parameters for the function
- y is the data to approximate
- f is an object function defining the gradient and the Hessian if needed
  It takes two parameters when called, the values x where the function should be computed and an array params that contains the parameters. The gradient of the function is the gradient for the parameters
```

Overrides: object.\_\_init\_\_

  


| Home | Trees | Indices | Help | PyDSTool |
| --- | --- | --- | --- | --- |

|  |  |
| --- | --- |
| Generated by Epydoc 3.0.1 on Fri May 4 15:24:09 2012 | http://epydoc.sourceforge.net |
